# The adenosinergic machinery in cancer: In-tandem insights from basic mechanisms to therapy

**DOI:** 10.3389/fimmu.2023.1111369

**Published:** 2023-02-23

**Authors:** Chifei Kang, Luyu Liu, Chengyu Wu, Lingyun Li, Xiao Jia, Wendi Xie, Siyu Chen, Xinying Wu, Huaxiao Zheng, Jingxin Liu, Rongsong Li, Bin Zeng

**Affiliations:** ^1^ College of Pharmacy, Shenzhen Technology University, Shenzhen, China; ^2^ College of Health Science and Environmental Engineering, Shenzhen Technology University, Shenzhen, China; ^3^ Guangdong Institute of Intelligence Science and Technology, Hengqin Guangdong-Macao In-Depth Cooperation Zone, Zhuhai, Guangdong, China; ^4^ Research Centre of Printed Flexible Electronics, School of Materials Science and Engineering, Harbin Institute of Technology, Shenzhen, China

**Keywords:** adenosine, machinery and mechanisms, cancer, therapy, EADO

## Abstract

Extracellular adenosine (eADO) signaling has emerged as an increasingly important regulator of immune responses, including tumor immunity. eADO is mainly produced from extracellular ATP (eATP) hydrolysis. eATP is rapidly accumulated in the extracellular space following cell death or cellular stress triggered by hypoxia, nutrient starvation, or inflammation. eATP plays a pro-inflammatory role by binding and activating the P2 purinergic receptors (P2X and P2Y), while eADO has been reported in many studies to mediate immunosuppression by activating the P1 purinergic receptors (A1, A2A, A2B, and A3) in diverse immune cells. Consequently, the hydrolysis of eATP to eADO alters the immunosurveillance in the tumor microenvironment (TME) not only by reducing eATP levels but also by enhancing adenosine receptor signaling. The effects of both P1 and P2 purinergic receptors are not restricted to immune cells. Here we review the most up-to-date understanding of the tumor adenosinergic system in all cell types, including immune cells, tumor cells, and stromal cells in TME. The potential novel directions of future adenosinergic therapies in immuno-oncology will be discussed.

## Introduction

**Figure 1:**
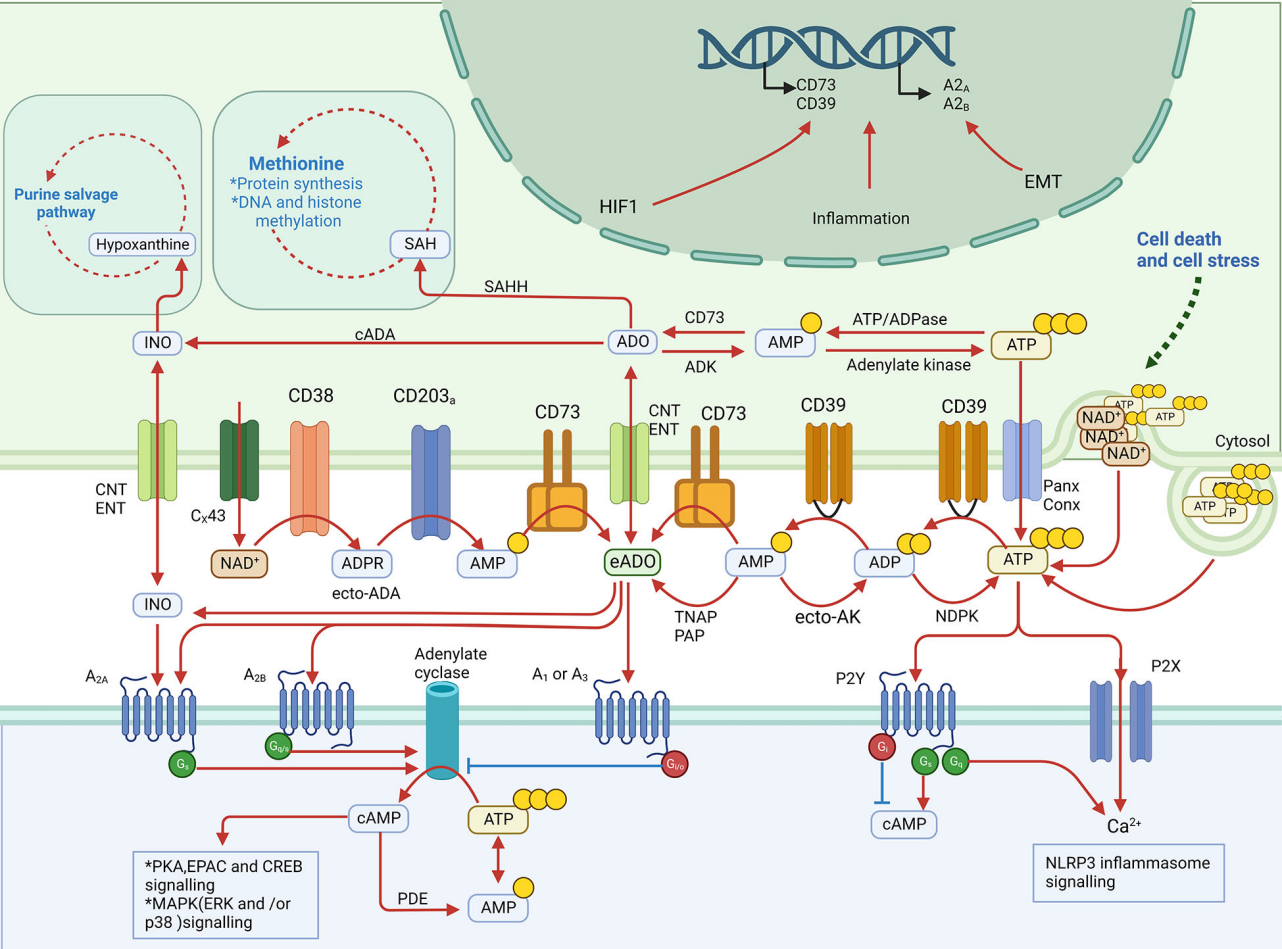
eADO metabolic pathways: production, degradation, and signaling.

Adenosine (ADO) is a metabolic intermediate involved in the ATP catabolism pathway and the synthesis of some important signaling molecules, such as cyclic adenosine monophosphate (cAMP) (1). Extracellular nucleotides, including purines and pyrimidines, have been unequivocally reported as signaling molecules involved in several systems such as blood pressure regulation, platelet activation, cardiovascular system remodeling, neurotransmission, anti-cell death, promotion of cell growth, and immunoregulation (2). Under physiological conditions, both ATP and ADO are usually at low levels in the extracellular space (3). Several cell conditions and stresses like cell membrane damage, ischemia, inflammation, and cancer could trigger the massive release of endogenous ATP in controlled manners such as regulated vesicular exocytosis and ion channel/transporter-mediated release but also in a direct cell-lytic way through cell destruction (**Figure 1**) (4–6). Thus, the accumulation of extracellular ATP (eATP) actually functions as a danger sign or nominated Danger-Associated Molecular Pattern (DAMP) to attract phagocytic cells to immigrate to the inflammatory sites and caution the whole immune system about the presence of pathogen-associated molecules and cell/tissue damage (7, 8). The activation of inflammation achieved by eATP is notably mediated through P2 purinergic receptors, including ligand-gated receptors (P2X) and metabotropic nucleotide-selective receptors (P2Y) (9, 10). Most family members of P2Y receptors promote oncogenic processes directly in tumor cells, while P2Y receptors in immune cells regulate these processes indirectly (11). Recent studies suggested that eATP activates P2X purinoceptor 7 (P2X7) expressed on macrophages, dendritic cells (DCs), granulocytes, T cells, and B cells to promote the formation of the NLRP3 inflammasome and the release of inflammatory cytokines such as IL-1β and IL-18 to enhance anti-tumor immunity (12–14). However, eATP is rapidly hydrolyzed to extracellular adenosine (eADO) in the tumor microenvironment (TME) since solid tumors normally have higher levels of ectonucleotidases than non-tumor tissues (15, 16).

eADO is primarily derived from the sequential hydrolysis of eATP mediated by several established ectonucleotidases (5). In a canonical route, eATP is hydrolyzed to extracellular ADP and AMP sequentially by CD39, which is known as ectonucleoside triphosphate diphosphohydrolase 1, and AMP is finally hydrolyzed to eADO by CD73, which is known as 5′-nucleotidase (17). However, the fate of eAMP is not limited to producing eADO; eAMP can also be phosphorylated sequentially to eATP by secreted or membrane-associated adenylate kinase (ecto-AK) and nucleoside diphosphate kinase (NDPK) (18).

The non-classical eADO production pathway is mediated by CD38, which is known as NAD^+^ ectohydrolase, and CD203a, which is known as ectonucleotide pyrophosphatase (19). Extracellular nicotinamide dinucleotide (NAD) released *via* gap junction protein connexin 43 (Cx43) regulation can be hydrolyzed to nicotinamide and ADP-ribose (ADPR) by CD38 (20, 21). Then CD203a consumes the ADPR to generate inorganic pyrophosphate and AMP, which are hydrolyzed by CD73 to eADO as mentioned above (19). In addition to CD73, prostatic acid phosphatase (22) and tissue-non-specific alkaline phosphatase (TNAP) were reported to hydrolyze eAMP to eADO (23, 24).

Analogous to eATP, in the extracellular space, the half-life of eADO is very short. The eADO molecule can be catalyzed directly into inosine by adenosine deaminase (ADA) and then into hypoxanthine by purine nucleoside phosphorylase (PNP) on the cell surface (25). eADO could also be transported into cells *via* concentrative nucleoside transporters (CNT1/2) or equilibrative nucleoside transporters (ENT1/2) (26). Inside cells, adenosine also has several metabolic pathways. The fundamental route is that intracellular ADO is phosphorylated by cytosolic adenylate kinase (ADK) to AMP, followed by conversion to ATP (27). Intracellular ADO could also be converted by cytosolic ADA (cADA) into inosine or by S-adenosyl-homocysteine hydrolase (SAHH) into S-adenosyl-homocysteine (SAH) involved in the methionine cycle (28). In conclusion, the eATP–CD39–CD73 pathway is the fundamental factor determining the concentration of eADO, but alternative ecto-enzymes also regulate metabolism, counteracting ATP-regenerating regulation.

Although the half-life of eADO is short, the concentration of eADO could remain high in TME. Cancer cell death due to rapid growth or chemotherapy contributes to ATP release and then eADO accumulation in the extracellular space (29). In addition to cancer cells, Treg cell deaths also provide ATP and CD39/CD73 to supply eADO production for immunosuppression in TME (30). Other than immune cells, cancer-associated fibroblasts (CAFs) in TME were reported to highly express CD73 induced by A_2B_ receptor activation to sustain a high level of eADO concentration in colorectal cancer (31). Under physiological conditions, ADO plays a role in balancing the immune system’s activation and overreaction. However, in TME, all cell types are also regulated by adenosine signaling and involved in eADO production, which ultimately builds up the role of eADO as a tumor cell growth supporter.

## Adenosine receptor pathways

eADO has its own specific receptors, which are P1 purinergic receptors. The P1 receptor family is composed of four G protein-coupled receptors: A_1_, A_2A_, A_2B_, and A_3_ (15, 32). These four receptors have different affinities for eADO. According to affinity, they can be roughly divided into two groups: A_1_, A_2A_, and A_3_ have affinities for eADO in the nanomolar range (100–310 nM), while A_2B_ has a comparatively low affinity for eADO in the micromolar range (15 µM) (33). The common primary function of P1 receptor family members is to regulate adenylate cyclase activity, which means modulating the intracellular cAMP concentration (34). A_1_ and A_3_, which are Gi/o(Gi/Go)-coupled adenosine receptors, implement inhibition of adenylate cyclase to decrease the intracellular level of cAMP. In contrast, A_2A_ and A_2B_, as Gq/s(Gq/Gs)-coupled adenosine receptors, increase the intracellular level of cAMP, which could potently dampen the immune response in some immune cells (35). A_2A_ receptor is generally expressed on most immune cells—monocytes, macrophages, DCs, neutrophils, natural killer (NK) cells, T cells, and natural killer T (NKT) cells; meanwhile, A_2B_ receptor is primarily highly expressed on macrophages and DCs (7).

**Figure 2:**
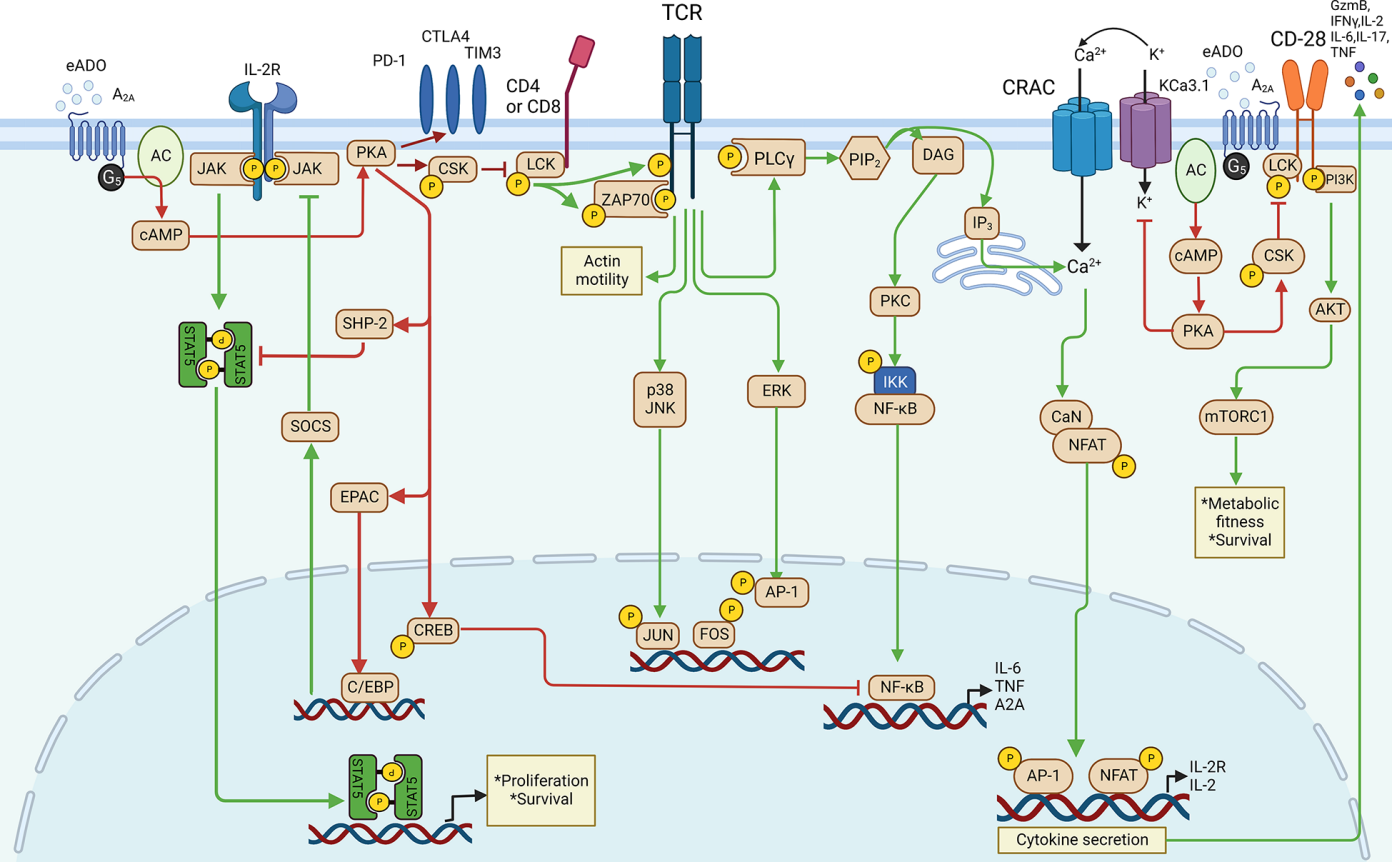
eADO/adenosine signaling in T cell.

In T cells (**Figure 2**), the pioneering work that provided evidence on the role of A_2A_-mediated immunosuppression in cancer can be traced to 20 years ago (36, 37). eADO binds to the A_2A_ receptor to stimulate the accumulation of cAMP, leading to the activation of the cAMP-dependent protein kinase A (38) signaling pathway, which negatively regulates the activation of T-cell receptor (TCR)-dependent transmembrane signaling *via* providing an OFF signal to activated immune cells (36). In addition to the cAMP/PKA pathway, eADO receptors can also function through cAMP-independent pathways such as DAG/PKC, MAPK (ERK and/or p38), and PI3K/AKT/mTOR pathways (39). In T cells, the eADO-activated A_2A_ receptor signaling-cAMP/PKA cascade triggers the direct inhibition of TCR activation *via* non-receptor tyrosine kinase (CSK). In addition, CSK inhibits CD28-mediated PI3K/AKT/mTORC pathways to decrease T cell protein synthesis, proliferation, and survival (40). PKA also phosphorylates the cAMP response element binding protein (CREB) to dampen the transcription activity of TCR downstream NF-κB (41, 42). In addition, PKA could activate SHP-2 and EPAC to impair T cell IL-2 receptor downstream signaling by inhibiting STAT5 and JAK, respectively, to suppress T-cell activation, survival, proliferation, and cytokine production (43–45). PKA inhibits KCa3.1 potassium channels, which causes extracellular Ca2+ cannot flux in through the calcium release-activated channels (CRAC) to suppress the upregulation of NFAT regulated genes which encode factors such as granzyme B (GzmB), IFNγ, TNF, IL-6, IL-17, IL-2, and IL-2R which are crucial to T-cell function and expansion (46). A_2A_ receptor activation was also reported to upregulate the expression of T-cell suppressive receptors such as programmed cell death protein 1 (PD-1), cytotoxic T lymphocyte antigen 4 (CTLA4), and T cell immunoglobulin and mucin domain-containing protein 3 (TIM3) so that T-cell immunosuppression is potentially enhanced (47, 48).

**Figure 3:**
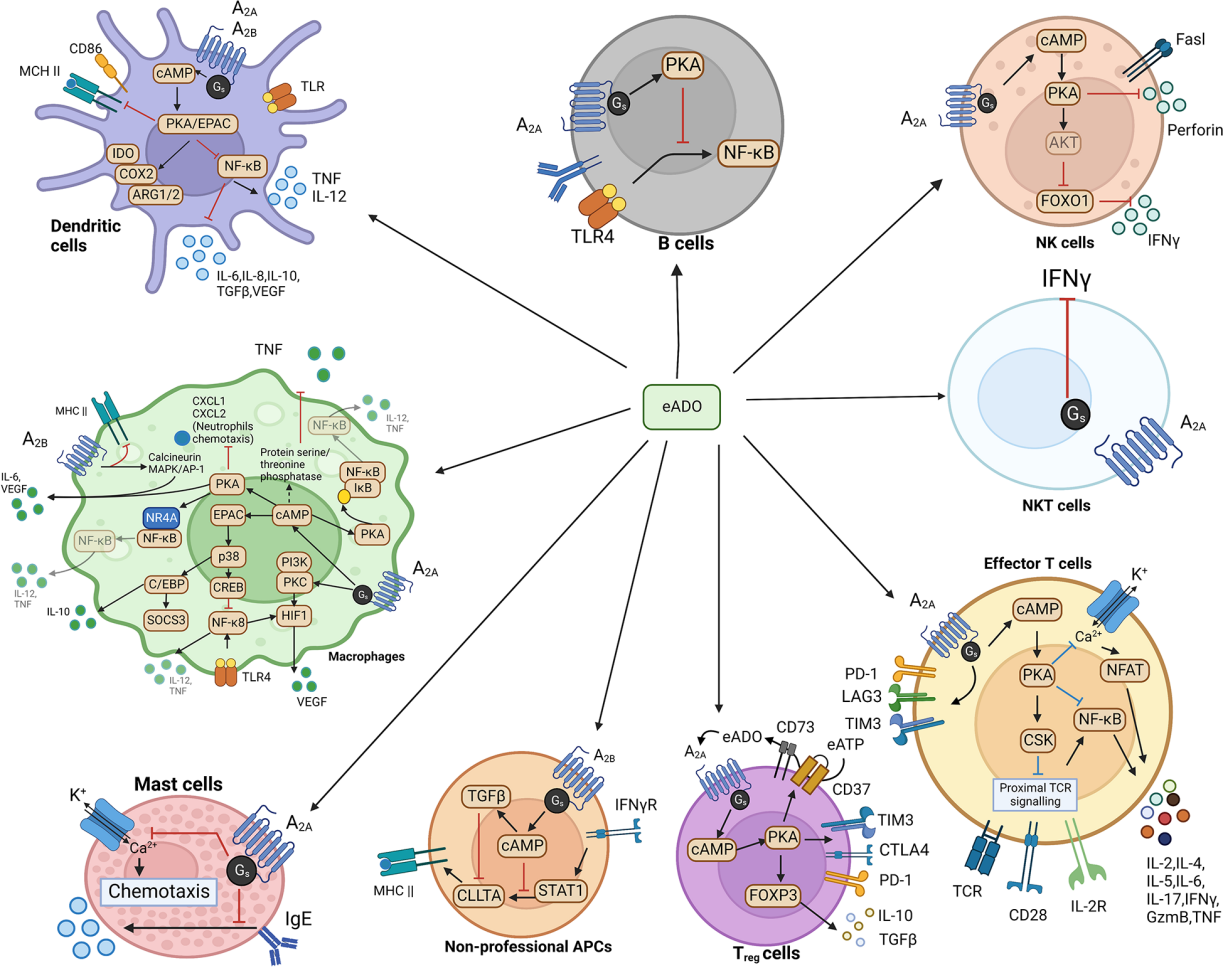
eADO/adenosine signaling in various immune cells.

In other immune cells (**Figure 3**), such as B cells, NF-κB, the downstream factor of the B cell receptor (BCR), and Toll-like receptor 4 (TLR4), are suppressed by PKA from activated A_2A_ receptor signaling, hence disrupting B cell survival (49). PKA from A_2A_ receptor signaling decreases production of IFNγ and perforin, which is the Fas ligand, to dampen the maturation and activity of NK cells (50, 51). A_2A_ receptor activation reduces IFNγ production in NKT cells and inhibits NKT cell activation (52). In non-professional antigen-presenting cells (APCs), such as fibroblasts, A_2B_ receptor-induced cAMP can suppress IFNγ-stimulated STAT1 activity and inhibit CIITA through upregulating TGFβ. The combined effects of this A_2B_ receptor signaling lead to a decrease of MHC II transcription, which attenuates tumor immune response (53). In macrophages, the expression level of both A_2A_ and A_2B_ receptors is promoted by Toll-like receptor signaling (54, 55). Activation of both A_2A_ and A_2B_ receptor signaling favors the shift of macrophages towards a tolerogenic tumor-promoting “M2” phenotype polarization accompanied by increased production of immunosuppressive IL-10, IL-6, and VEGF as well as a decrease in pro-inflammatory IL-12 and THF (38, 56). Similarly, in dendritic cells (DCs), both A_2A_ and A_2B_-mediated cAMP/PKA signaling enhance the production of IL-10, IL-6, VEGF, and TGFβ plus indoleamine 2,3-dioxygenase (IDO), cyclooxygenase 2 (COX2), and arginase 1/2 (ARG 1/2), which meanwhile dampen the expression of IL-12 and TNF (57). Based on most currently known data, in a sense, the A_2A_ receptor elicits immunosuppression in both lymphocytes and myeloid cells. In contrast, the A_2B_ receptor elicits immunosuppression mainly from myeloid cells.

*In cancer cells:* In the extracellular space of solid tumor TME, the concentration of eATP is considerably high due to both the passive release from tumor cell necrosis and the active secretion from tumor cells and other TME cells. Many factors, such as mechanical stress, starvation, hypoxia, and chronic inflammation, could induce this active secretion of ATP (12, 58). eATP binds to both P2X and P2Y, notably P2X7 expressed in immune cells in TME such as DCs, macrophages, B cells, and T cells (6, 12). The activation of P2X7 could promote calcium influx to enhance NLRP3 inflammasome formation, leading to antitumor immunity promotion (29, 59). In this regard, TME seems to provide a strategy to promote the inflammatory response, which could potentially contribute to antitumor activity. In fact, tumor cells express a higher level of ectonucleotidases such as CD39 and CD73 to execute the hydrolysis of eATP to eADO. In addition to the most reported transcription factor, hypoxia-inducible factor 1 (HIF1), several proteins such as TGFβ, TNF, IL-2, and IL-6 could enhance the expression of CD39 and CD73 (16, 60). As mentioned above, CD39 and CD73 are also generally expressed in immune cells; thus, ectonucleotidases from both tumor cells and immune cells together produce a large amount of eADO in the TME niche.

HIF1 as a transcription factor was found to increase the expression of CD39, CD73, A_2A_, and A_2B_ as well as suppress the expression of both ENTs and adenylate kinase, leading to eADO accumulation in solid tumor TME, which is normally hypoxic (61–66). The upregulation of CD39, CD73, and A_2B_ in various cancers was reported to positively correlate with poor prognosis in patients (60, 67). In particular, there have already been tremendous studies showing that high expression of CD39 and CD73 consistently correlated with poor prognosis in patients with those high incidence rates and malignant cancers such as ovarian, gastric, rectal, breast cancers (including TNBC), hepatocellular carcinoma (HCC), and non-small-cell lung cancers (NSCLCs) (68–73). This is the rationale that supports many current and ongoing clinical trials targeting CD39 and CD73.

More studies uncovered the molecular mechanisms involved in CD73 upregulation in cancer cells in addition to the regulation of HIF1 and TGFβ. Epithelial-to-Mesenchymal Transition (EMT) factors such as WNT/β-catenin pathway activators and TWIST were found to upregulate the expression of CD73 in human tumors (74). Mutations or upregulation of TP53, KRAS, BRAF, and EGFR also positively correlated with increased expression of CD73 in various human tumors (73, 75–77). In tumor cells, especially those with an EMT phenotype, CD73 and some factors like TGFβ form a positive feedback loop in that TGFβ signaling increases CD73 expression and CD73 produces more eADO stimulating A_2A_ and A_2B_ receptor pathways to favor TGFβ production and secretion; thus, CD73/eADO receptor signaling contributes to EMT promotion in cancer cells (78). Since the high concentration of extracellular NAD^+^ is present in the TME niche in some cancer types, probably due in part to the altered metabolism in cancer cells, the non-classical eADO production pathway mediated by CD38 also plays an influential role in eADO signaling in several solid tumors (22, 79).

The effects of eADO are not limited to immune cells to implement immunosuppression but also on cancer cells directly to regulate tumor proliferation, growth, anti-apoptosis, and metastasis. The PI3K/AKT/mTORC signaling pathway could be promoted upon eADO-mediated A_2A_ receptor signaling to promote cell proliferation, tumor progression, and metastasis in melanoma, hepatocellular carcinoma, and gastric cancer (80–82). The A_2B_ receptor was found to stimulate different downstream signaling compared to A_2A_ in cancer cells. In TNBC cells, activation of the A_2B_ receptor occurs notably *via* the ERK1/2-MAPK pathway. Knockdown of the A_2B_ receptor in TNBC cells suppresses cancer cell proliferation and lung metastasis (67). A_2B_ receptor signaling could activate FOS-related antigen 1 (FRA-1) and the small GTPase RAP1B to enhance TNBC cells’ lung metastasis in mouse models (83, 84). An intriguing finding is that A_2B_ receptor signaling is constitutively activated in prostate cancer cells to promote cancer cell proliferation *in vitro*. However, activation is not dependent on the availability of the A_2B_ receptor ligand, eADO. This study suggested potential adenosine-independent signaling under the A_2B_ receptor in cancer cells (85). The EMT process has an unequivocal interaction with adenosine signaling. Enhancing EMT levels leads to increased CD73 expression and thus eADO receptor signaling, which in turn promotes the EMT process in ovarian cancer (68, 78). Cancer cells with an EMT phenotype usually exhibit cell stemness, which is suggested as a potential cancer stem cell. In breast cancer and glioblastoma, hypoxia-induced A_2B_ receptor activation results in the maintenance of self-renewing tumor cells in the mouse model (86, 87). In a hepatocellular carcinoma study, CD73 was found to be upregulated, leading to A_2A_ receptor activation, which results in cancer cells’ EMT and stemness promotion through increasing SOX9 expression and activity (88).

## Therapy for cancer targeting adenosine signaling pathway

Not surprisingly, drugs designed to target the adenosine signaling pathway have been blooming vigorously for the last decade. Strategies for targeting adenosine signaling pathway could generally be classified into two groups: ① inhibition of adenosine production and prevention of ATP degradation simultaneously in TME *via* targeting CD73 and/or CD39; and ② interruption of adenosine signaling through blocking A_2A_ and A_2B_ receptors. According to ongoing pre-clinical research and clinical trials, drugs targeting the CD73 and A_2A_ receptors are the mainstream adenosine pathway inhibitors. Most CD73 inhibitors are monoclonal antibodies for potential pharmacological application, whereas small-molecule inhibitors are currently the only available clinical drugs targeting A_2A_ and A_2B_ receptors since they are G protein-coupled receptors (GPCRs) with specific conformations notoriously difficult for antibody binding.

*Targeting A2A and/or A2B in cancer:* A_2A_ antagonists were initially developed for neurological disorders such as Parkinson’s disease or adult attention deficit hyperactivity disorder (ADHD) (89, 90). Their evaluations in clinical trials suggested a great tolerability and safety profile. The available preliminary data of several A_2A_ antagonists in clinical trials with cancer patients showed good tolerability and exhibited some effects. They are CPI-444 (Corvus), PBF-509 (Novartis/Pablobiofarma), EOS100850 (iTeos), MK-3814 (Merck), AZD4635 (AstraZeneca/Heptares), and a dual A_2A_ and A_2B_ antagonist AB928 (Arcus) (91–96). PBF-1129 (Pablobiofarma), a selective A_2B_ antagonist, has also been developed and is being tested in a clinical trial involving NSCLC cancer patients. In two clinical trials, CPI-444 was administered alone and in combination with Atezolizumab (PD-L1 antibody, Genentech) in patients with renal and advanced metastatic castration-resistant prostate cancer (91). Most common adverse events are in grades 1–2, including fatigue, pruritus, nausea, diarrhea, rash, vomiting, and anemia as well as several in grades 3–4, such as decreased appetite, anemia, arthralgia, and peripheral edema. A better outcome (median progression-free survival of 5.8 months versus 4.1 months and overall survival of 90% versus 55% at 20 weeks) was observed with the A_2A_ antagonist CPI-444 plus the anti-PD-L1 antibody atezolizumab compared to CPI-444 alone in patients with advanced-stage renal cell carcinoma (91). Similar results have been reported in patients with mCRPC: 57% of patients (eight of 14) experienced disease control, with five partial responses and two stable disease responses.

*Targeting CD73 and/or CD39:* There are several anti-CD73 monoclonal antibodies in phase I/II clinical trials currently, including MEDI9447 (MedImmune), BMS-986179 (BMS), NZV930 (Novartis), and CPI-006 (Corvus), as well as a small molecule inhibitor, AB680 (Arcus) (97–99). In these clinical trials, CD73 inhibitors were administered alone and in combination with PD-1/PD-L1 monoclonal antibodies. Most adverse events were mild, and most outcomes indicated a decreased primary tumor expansion rate, less metastasis formation, and an improved survival rate (99). In addition to CD73, monoclonal antibodies and small-molecule antagonists to CD39 and CD38 are also under development (22, 99).

Targeting drugs are listed in **Table 1**.

**Table 1:** Representative eADO pathway-targeting drugs which were involved in the most recent clinical trials.

Target	Cancer Type	Drug Name	Company
A**_2A_** receptor	Advanced solid tumors, non-Hodgkin lymphoma	CPI-444	Corvus
A**_2A_** receptor	Non-small cell lung cancer	PBF-509	Novartis/Pablobiofarma
A**_2A_** receptor	Adult solid tumor	EOS100850	iTeos
A**_2A_** receptor	Advanced solid tumors	MK-3814A	Merck
A**_2A_** receptor	Advanced solid tumors	AZD4635	AstraZeneca/Heptares
A**_2B_** receptor	Non-small cell lung cancer	PBF-1129	Pablobiofarma
A**_2A_** and A**_2B_** receptors dual antagonist	Metastatic castrate resistant prostate cancer	AB928	Arcus
CD73	Solid tumors	MEDI9447	MedImmune
CD73	Advanced solid tumors	BMS-986179	BMS
CD73	Advanced solid tumors	NZV930	Novartis
CD73	Advanced solid tumors, non-Hodgkin lymphoma	CPI-006	Corvus
CD73	Healthy volunteers	AB680	Arcus
CD38	Lymphoma Prostate, Non-small cell lung cancer	Isatuximab/SAR650984	Sanofi

## Cautions in the adenosine targeting therapy

*The existing controversial effects of adenosine blockage in cancer:* The prevalent view is that eADO production and eADOA/ARs signaling activation are associated with poor clinical outcomes. However, it is not substantial for every type of cancer. A group found that in endometrial carcinoma, CD73 played a critical role in tumor suppression (100), whereas another group reported that in endometrial carcinoma, the loss of CD73 is essential for tumor progression (101). Although several studies found a link between A_2A_ expression or activation and poor outcomes in breast cancer, Vasiukov et al. revealed a positive correlation between A_2A_ receptor gene expression and better survival data in basal-type breast cancer and TNBC patients (102). In addition, adenosine receptors (ARs) also exhibit both stimulatory and inhibitory effects in melanoma (80). A similar contradictory effect of adenosine receptors on hepatocellular carcinoma progression has also been reported (103). More mechanisms and pre-clinical studies are necessary to provide fundamental knowledge for adenosine targeting therapy.

*Specificity issue in adenosine receptor blockage:* As mentioned, adenosine receptors are members of the GPCR family. The conformational complexity of GPCR gives rise to the difficulty of developing antibodies to target the receptors. The currently available pharmacological inhibitors of ARs are small molecules that have the notorious disadvantage of engaging of multiple targets (poly-pharmacology). Several compounds, which were previously confirmed as binding interactors of A_1_, A_2_, and A_3_ receptors, were found to have intracellular binding targets (104, 105). In addition, the putative selective A_2B_ receptor agonist BAY 60-6583 was reported to have other binding molecules to increase CAR-T cell activity independently of the A_2B_ receptor (106).

There is still a large amount of work to be done to pursue better safety and efficacy in adenosine signaling targeting therapy.

## Conclusion

Both eATP and eADO are important signal molecules in the physiological processes of cells and tissues. Tissue damage or various cell stresses such as hypoxia, starvation, and mechanical stress, which are common in the TME niche, could stimulate eATP accumulation and rapid hydrolysis to eADO. This would lead to dramatically increased eADO. This eATP–eADO metabolic pathway is involved in pathological shifts in several aspects: rapid eATP degradation dampens the inflammatory response; accumulation of eADO triggers immunosuppression; and it promotes tumor cell proliferation and EMT.

In adenosine signaling, pre-clinical studies suggested the CD39–CD73–A_2A_ receptor pathway is an attractive and tractable therapeutic target for cancer treatment. Inhibitors targeting the CD73 and A_2A_ receptors exhibited good tolerability and achieved some therapeutic effects in some clinical trials. However, several knowledge gaps are worthy of exploring to assist further pre-clinical and clinical trial design (1): What are the potential compensation pathways for the inhibition of eADO signaling? They are probably not limited to intracellular ADO release and ADO-independent adenosine receptor activation. (2) More combined therapies, such as immune checkpoint blockers and adenosine signaling inhibitors, have shown better efficacy. (3) What are reliable biomarkers to indicate which patient subgroups have a higher chance of benefiting from treatments targeting eADO signaling? In conclusion, the adenosinergic system offers new therapeutic strategies aimed at limiting immunosuppression and potentiating antitumor immune responses.

## Author contributions

BZ, RL, and JL, carried out the concepts, design, definition of intellectual content, literature search. CK and LLiu contributed writing and editing the manuscript. LLi, XJ, and HZ helped in formatting the manuscript. CW helped perform the manuscript with constructive discussions. WX, SC, and XW carried out the figures in manuscript. All authors contributed to the article and approved the submitted version.
